# Antifungal Activity of Tea Tree (*Melaleuca alternifolia* Cheel) Essential Oils against the Main Onychomycosis-Causing Dermatophytes

**DOI:** 10.3390/jof10100675

**Published:** 2024-09-27

**Authors:** Esther Mingorance Álvarez, Julia Villar Rodríguez, Olga López Ripado, Raquel Mayordomo

**Affiliations:** 1 Department of Physiology, University Centre of Mérida, University of Extremadura, 06800 Mérida, Badajoz, Spain; emingorance@unex.es; 2 Department of Nursing, Physiotherapy and Occupational Therapy, Faculty of Health Sciences, University of Castilla la Mancha, 45600 Talavera de la Reina, Toledo, Spain; julia.villar@uclm.es; 3 Department of Anatomy, Cellular Biology and Zoology, University Centre of Plasencia, University of Extremadura, 10600 Plasencia, Cáceres, Spain; olga@unex.es

**Keywords:** antifungal activity, clinical isolates, dermatophytes, essential oil, fungal infection, onychomycosis

## Abstract

Onychomycosis is a common fungal infection that affects the nails and accounts for approximately 50% of all nail diseases. The main pathogens involved include dermatophytes, such as *Trichophyton rubrum*, members of the *T. mentagrophytes* complex, and emerging pathogens in this infection, *T. schoenleinii* and *T. tonsurans*. Tea tree (*Melaleuca alternifolia* Cheel) essential oil (EO) has been proposed as a promising natural alternative to traditional treatments due to its antimicrobial properties. Among its more than 100 compounds, terpinen-4-ol is one of the main contributors to the antifungal action of this EO. To determine the antifungal activity of tea tree EO against dermatophytes, we designed an *in vitro* study using EUCAST-AFST protocols to obtain the values of MIC (minimum inhibitory concentration) and MFC (minimum fungicidal concentration) of several commercial *M. alternifolia* Cheel EOs against three species of dermatophytes isolated from clinical samples with suspected toenail onychomycosis. The results showed that the microorganism most sensitive to the action of the EO was *T. rubrum*, which had an MIC value more than 13 times lower than the value obtained for *T. schoenleinii* (0.4% *v*/*v*), the most resistant isolate. No differences in antifungal activity were observed by the analysed EOs or between the MIC and MFC values. These *in vitro* results suggest that tea tree EO is a viable option for the alternative treatment of onychomycosis, although clinical studies are needed to confirm the long-term antifungal activity, safety and efficacy of the oils studied in a clinical context.

## 1. Introduction

Onychomycosis is a fungal infection affecting the nails, and one of the most common nail disorders. It is thought to account for approximately 50% of all nail diseases and has an estimated global prevalence of around 5.5% [1,2,3]. The microorganisms most frequently associated with onychomycosis include dermatophytes, such as *Trichophyton rubrum,* and members of the *T. mentagrophytes* complex, and yeasts of the genus *Candida* and non-dermatophyte moulds, such as *Scopulariopsis brevicaulis* and *Aspergillus* spp. [4,5,6,7,8]. In the genus *Trichophyton*, the species *T. schoenleinii* and *T. tonsurans* [9,10,11,12] are frequently identified. Both of these are responsible for tinea capitis (scalp ringworm), as they are able to colonise and degrade keratinised tissues of the human body [13].

Nail infections can cause nail deformities, pain, and discomfort [14,15]. Onychomycosis has a considerable psychological impact on self-image and can affect self-esteem [16]. It is also hard to treat because of the keratinic nature of the infected tissue, which is difficult for antifungal agents to penetrate [17].

In the search for effective and natural novel treatments against onychomycosis, tea tree (*Melaleuca alternifolia* Cheel) essential oil (EO) has proven to be a promising solution. It has proven antimicrobial properties, and its use is supported by growing scientific evidence [18,19,20,21]. Although several species of the genus *Melaleuca* are suitable for this type of treatment, *M. alternifolia* Cheel is the most frequently used because of the high concentration of terpinen-4-ol in its EO [22,23], one of the main reasons for its antifungal activity [24]. Another reason for the frequent use of this EO is its efficacy against a wide range of microorganisms, including those that cause onychomycosis [25,26]. This compound, together with alpha-terpineol and eucalyptol (also known as 1.8-cineole), are known to increase the cell membrane permeability of fungi and alter mycelial morphology and cell ultrastructure [27].

The marketing and composition of tea tree EO are regulated by international standards. ISO 4730:2017/Amd 1:2018 [28] states the requirements for *Melaleuca* spp. EO and determines the quality criteria for its sale, including the chemical composition, impurity levels and physical parameters.

Several studies have analysed the antifungal activity of *M. alternifolia* Cheel EO and established the values of MIC (minimum inhibitory concentration) and MFC (minimum fungicidal concentration) against various dermatophytes [18,24,29,30], using reference strains of the main infectious agents [31,32]. However, little information is available about clinical isolates obtained during examination of the feet. The aim of this study is to determine the MIC and MFC of three commercial *M. alternifolia* Cheel EO against clinical isolates of the main causative agents of onychomycosis of the genus *Trichophyton*. This will help to define the initial procedures before clinical trials are conducted to assess the potential of EO therapies as alternative or complementary treatments to traditional antifungal approaches.

## 2. Materials and Methods

### 2.1. Origin and Composition of Essential Oils

Three commercial *M. alternifolia* Cheel EO (Esencias Lozano^®^, Naissance^®^ and Marnys^®^) were used. The three companies are based in Europe and have online stores: Esencias Lozano^®^ in Caravaca de la Cruz, Spain; Naissance^®^ in Neath, United Kingdom; and Marnys^®^ in Cartagena, Spain.

Essential oil composition was analysed using the information provided by the commercial brands. All EO had a terpinene-4-ol percentage higher than 40%, and the compounds alpha-terpineol and eucalyptus did not exceed 4.41% (Table 1). All concentrations were within the ranges defined by ISO 4730:2017/Amd 1:2018 [28] and were indicated in the product information sheets.

### 2.2. Microbial Strains and Inoculum Preparation 

The clinical isolates used were obtained during examination of the feet of three patients with suspected onychomycosis and handled at the University Centre of Plasencia. The study comprised samples taken from three women with average age 55.7 years (±7.4 years) and mean body mass index 25.5 kg/m^2^ (±2.4 kg/m^2^). Participants had no history of toenail fungal infection and no concomitant illnesses. 

Toenail tissue samples were taken following Pérez Pico et al. [33]. Samples were observed under conventional light microscopy with 30% (*p*/*v*) KOH solution (Labbox, Barcelona, Spain) to determine the presence of dermatophytes. The species of *Trichophyton* were identified using lactophenol blue solution (Labbox, Barcelona, Spain), following [34,35]. Microbiological cultures of each sample were grown in triplicate in Sabouraud dextrose agar selective medium with chloramphenicol and cycloheximide (Condalab, Torrejón de Ardoz, Spain), incubated at a constant temperature of 25–28 °C for 2–6 weeks. 

The *T. schoenleinii, T. tonsurans,* and *T. rubrum* isolates were subcultured in Sabouraud dextrose agar (Condalab, Madrid, Spain) supplemented with cycloheximide 300 mg/L and chloramphenicol 50 mg/L and incubated 7–10 days at 30 °C to adequate sporulation.

### 2.3. Antifungal Susceptibility Testing 

The MIC values were determined following the protocols of the European Committee on Antimicrobial Susceptibility Testing Subcommittee on Antifungal Susceptibility Testing (EUCAST-AFST) for dermatophytes [36]. The EO concentrations analysed were from 0.01% (*v*/*v*) to 1.25% (*v*/*v*), dissolved in dimethyl sulfoxide (DMSO; PanReac AppliChem, Barcelona, Spain) at a final concentration of 2% (*v*/*v*). The RPMI 1640 culture medium with L-glutamine (Sigma-Aldrich, Taufkirchen, Germany) supplemented with 2% (*w*/*v*) glucose (PanReac AppliChem, Barcelona, Spain) and buffered with 3-(N-morpholino) propanesulfonic acid (MOPS; Sigma-Aldrich, Taufkirchen, Germany) was inoculated with a suspension adjusted to 2–5 × 10^5^ CFU/mL, supplemented with cycloheximide 300 mg/L and chloramphenicol 50 mg/L (PanReac AppliChem, Barcelona, Spain). Microdilutions were incubated for 7 days at 30 °C without agitation until they were read. MIC was established as the lowest EO concentration in mg/L that inhibited fungal growth [36]. 

**Figure 1:**
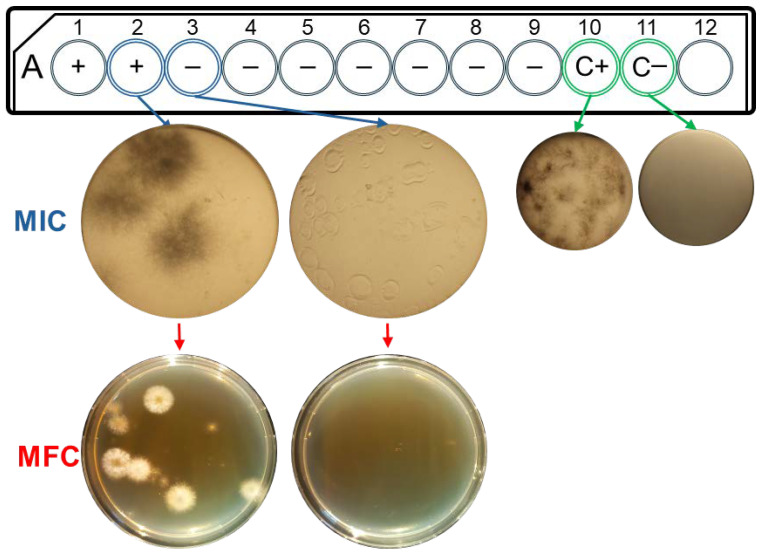
Antifungal susceptibility testing of *T. tonsurans* against Esencias Lozano^®^ *M. alternifolia* Cheel tea tree EO. A: diagram of microdilution plates with well numbers (1-12); +: with fungal growth; −: without fungal growth; C+: positive growth control; C−: negative growth control. MIC: minimum inhibitory concentration in well microdilution plate. Images taken at 2.5× for greater detail. MFC: minimum fungicidal concentration in Petri dishes.

To determine MFC, 10 µL from wells without growth was inoculated onto Sabouraud dextrose agar medium (Condalab, Madrid, Spain), supplemented with cycloheximide 300 mg/L and chloramphenicol 50 mg/L, and incubated for 2 days at 30 °C [37,38]. Minimum inhibitory concentration was established as the lowest EO concentration without fungal growth (Figure 1). All experiments were performed independently in triplicate.

## 3. Results

### Antifungal Susceptibility Results

The antifungal activity of the three EO was analysed by determining the MIC and MFC values of the clinical isolates (Figure 1). All isolates were sensitive to EO concentrations lower than 0.5% (*v*/*v*). *T. schoenleinii* was the most resistant (0.4% *v*/*v*), and *T. rubrum* (0.03% *v*/*v*) was the most sensitive. Therefore, the microorganisms showed different responses to *M. alternifolia* Cheel tea tree EO. However, no differences were detected in MIC or MFC between the three commercial EOs tested, as they all showed the same result (Table 2).

## 4. Discussion

The results of this research demonstrate the *in vitro* efficacy of tea tree EO in inhibiting the growth of microorganisms that cause toenail onychomycosis, assessed by determining the MIC and MFC of three samples from clinical isolates. The antifungal activity observed can be attributed to certain compounds of the EO, especially terpinen-4-ol, alpha-terpineol, and eucalyptol, as described elsewhere [23,27,28]. All three commercial EOs analysed complied with ISO 4730:2017/Amd 1:2018 [28], which defines the amounts of the terpinen-4-ol component (not less than 35%) and eucalyptol (15% or more) in the EO. This standard also establishes a maximum and minimum range for other tea tree EO compounds to ensure that the product characteristics are homogeneous, safe, and good-quality. 

Studies by Roana et al. [21] and Hammer et al. [39] on the efficacy of *M. alternifolia* Cheel EO against *T. rubrum* in onychomycosis clinical samples reported 0.06–0.3% MIC and 0.06–0.25% MFC. In contrast, our study found 0.03% MIC and MFC, indicating a greater antifungal efficacy of the three EOs analysed. These discrepancies could reflect differences in the testing methodology, as the methods to determine antifungal activity differed between the three studies. They may also be explained by the chemotype of the EO used, especially the terpinen-4-ol concentration. Roana et al. [21] used a different concentration from ours, and Hammer et al. [39] did not indicate the concentration. These and other variables highlight the difficulty of obtaining comparable and reproducible results using different standards. The proven ability of the analysed EO to act at low concentrations would reduce the risk of side effects. These EOs could be an attractive choice for patients seeking treatments with fewer adverse effects, polymedicated patients, or those who are resistant or intolerant to synthetic antifungal treatments [40,41]. Because the literature does not mention the MIC and MFC of tea tree EO against clinical isolates of *T. schoenleinii* and *T. tonsurans*, the results reported in this study are a novel contribution.

The lack of differences detected in MIC and MFC among the commercial EO tested suggests homogeneity in the composition and quality of the products available on the market, which are subject to standardisation regulations [28]. This is crucial to ensure therapeutic efficacy in the use of tea tree EO to treat onychomycosis, provided that quality standards are maintained [28,42].

The higher resistance of *T. schoenleinii* and *T. tonsurans* compared to *T. rubrum* could be associated with differences in the cell wall and the structure of the membrane. The first two microorganisms are larger under the microscope and have a thicker, more partitioned cell wall than *T. rubrum*. These factors influence susceptibility to antifungal agents [43,44]. Efflux pumps identified in the plasmatic membrane of members of the *Trichophyton* genus are responsible for resistance to multiple antifungal therapies. In *T. rubrum*, differences in expression detected in function of the presence of antifungal agents could explain the differences in antifungal activity among species of *Trichophyton* [45]. Although *T. rubrum* is the dermatophyte most frequently associated with onychomycosis (8), *T. schoenleinii* and *T. tonsurans* are becoming more prevalent in toenail infections [46]. This suggests the adaptation of these species for infection, at least in toenails. Differences in geographical distribution between species of this genus must also be taken into account [47].

Commercial tea tree EO shows considerable potential as an alternative antifungal treatment for dermatophyte onychomycosis, with demonstrated efficacy *in vitro*. The results provide the information needed to commence the next stage of clinical trials to assess the activity of these commercial EOs *in vivo*. Clinical studies will determine the long-term safety and efficacy of the commercial EOs studied, both individually and in combination with other antifungal treatments. They will also help to define the formula for topical administration of the EO to provide a comprehensive, alternative solution for managing onychomycosis.

## Figures and Tables

**Table 1:** Concentration of the EO compounds with antifungal activity obtained by gas chromatography coupled with mass spectrometry according to the product information sheets.

Component	Naissence^®^	Marnys^®^	Esencias Lozano^®^
Terpinen-4-ol	41.90%	44.84%	40.76%
Alpha terpineol	n.d.	2.78%	4.41%
Eucalyptol	2.30%	4.23%	1.74%
Para Mentha-1,4-Diene	22.20%	n.d.	n.d.
Alpha Terpinene	10.90%	10.92%	10.10%
Gammaterpinene	n.d.	22.31%	20.23%
Alpha Terpinolene	3.0%	n.d.	n.d.
Terpinolene	n.d.	2.77%	3.17%
Alpha Pinene	3.90%	3.94%	2.17%
Beta Pinene	n.d.	0.38%	n.d.
Para Cymene	3.60%	2.72%	2.44%
Limonene	2.80%	2.67%	n.d.
Sabinene	0.20%	0.24%	n.d.
Aromadendrene	0.70%	2.13%	0.45%
Aromadendrene Isomer	n.d.	0.09%	n.d.
Allo Aromandendrene	n.d.	0.31%	n.d.
Cadinene	n.d.	0.01%	n.d.
Delta Cadineno	n.d.	n.d.	1.62%
Globulol	n.d.	0.24%	n.d.
Viridiflorol	n.d.	0.06%	n.d.
Para Ment-3-Ene	n.d.	0.08%	n.d.
Beta Myrcene	n.d.	0.07%	n.d.
Alpha Phellandrene	n.d.	0.15%	n.d.
Linalool	n.d.	0.03%	n.d.
Beta Caryophyllene	n.d.	0.17%	0.27%
Cis-P-Menth-2-En-1-Ol	n.d.	0.07%	n.d.
Cadina-3,5-Diene	n.d.	0.23%	n.d.
Zonarene	n.d.	0.04%	n.d.
Alpha Humulene + Cis-Piperitol	n.d.	0.05%	n.d.
Neral	n.d.	0.05%	n.d.
Ledene	n.d.	0.78%	n.d.
Alpha Muurolene + B-Selinene	n.d.	0.09%	n.d.
Geranial + A-Selinene	n.d.	0.14%	n.d.
Spathulenol	n.d.	0.00%	n.d.

^®^: trademark; %: percentage; n.d.: no data available.

**Table 2:** Antifungal activity of commercial *M. alternifolia* Cheel essential oils.

Strain	Units	Esencias Lozano^®^		Naissance^®^		Marnys^®^	
		MIC	MFC	MIC	MFC	MIC	MFC
*T. schoenleinii*	% (*v*/*v*)	0.4 3.582	0.4 3.582	0.4 3.582	0.4 3.582	0.4 3.582	0.4 3.582
	mg/L						
*T. tonsurans*	% (*v*/*v*)	0.09 805.95	0.09 805.95	0.09 805.95	0.09 805.95	0.09 805.95	0.09 805.95
	mg/L						
*T. rubrum*	% (*v*/*v*)	0.03 268.65	0.03 268.65	0.03 268.65	0.03 268.65	0.03 268.65	0.03 268.65
	mg/L						

^®^: Registered trademark; MIC: minimum inhibitory concentration; MFC: minimum fungicidal concentration.

## Data Availability

The original contributions presented in the study are included in the article, further inquiries can be directed to the corresponding author.
